# Midazolam Attenuates Esketamine-Induced Overactive Behaviors in Mice Before the Sedation, but Not During the Recovery

**DOI:** 10.3389/fvets.2022.829747

**Published:** 2022-04-11

**Authors:** Qinjun Chu, Meng Mao, Yafan Bai, Liwei Sun, Dongqing Zhang, Ping Zheng, Xiaogao Jin

**Affiliations:** ^1^Department of Anesthesiology and Perioperative Medicine, Zhengzhou Central Hospital Affiliated to Zhengzhou University, Zhengzhou, China; ^2^West Houston Family Practice, Houston, TX, United States; ^3^Metabolic Disease Research Center, Zhengzhou Central Hospital Affiliated to Zhengzhou University, Zhengzhou, China; ^4^Center for Advanced Medicine, College of Medicine, Zhengzhou University, Zhengzhou, China; ^5^Trauma Center of Henan Province, Zhengzhou Central Hospital, Zhengzhou, China

**Keywords:** esketamine, midazolam, anesthesia, behavior, hyperactivity introduction

## Abstract

Esketamine showed more potency, more rapid recovery from anesthesia, and less psychotomimetic side effects when compared with ketamine. However, the patients still experience psychotomimetic side effects of esketamine. In order to investigate whether midazolam can attenuate the esketamine-induced overactive behaviors and neuronal hyperactivities, midazolam 0, 40, 80, and 120 mg/kg combined with esketamine 50 mg/kg were administrated on Kunming mice to assess the behaviors changes during anesthesia. The indicators, including action time, duration of agitation before the sedation, duration of sedation, duration of loss of pedal withdrawal reaction (PWR), duration of loss of righting reaction (RR), duration of agitation during the recovery, and recovery time, were monitored for up to 3–4 h after intraperitoneal administration. The results demonstrated that midazolam 40, 80, and 120 mg/kg efficiently decreased the esketamine-induced overactive behaviors including ataxia, excitation, and catalepsy before sedation. Midazolam and esketamine synergically improved the anesthesia quality assessed by PWR and RR. However, even high doses of midazolam were not able to suppress the esketamine-induced psychotomimetic effects during the recovery.

## Introduction

Ketamine is an anesthetic of N-methyl-D-aspartic acid receptor (NMDA receptor) antagonist, which can produce unique dissociative anesthesia (1–3). Ketamine contains equal parts of the (*S*)-ketamine enantiomer (esketamine) and (*R*)-ketamine enantiomer (4). Esketamine has been regarded as the active stereoisomer due to its higher affinity (about three- to four-fold) for the NMDA receptor and greater anesthetic potency than (*R*)-ketamine and ketamine (4, 5). Now, esketamine is commercially available for anesthesia and exhibited 2–3 folds more potent than ketamine (6). Both ketamine and esketamine have dissociative side effects (5). The dissociative effects include loss of self, inability to move the body, and isolation of mind from the body (3). The symptoms are similar to schizophrenia, and called schizophrenia-like changes. The patient with esketamine may experience emergence phenomena which manifested floating sensation, vivid pleasant dreams, nightmares, hallucination, and delirium (2). However, compared to ketamine, esketamine demonstrated faster and smoother recovery, less psychotomimetic adverse effects, and less respiratory depression (2). Esketamine and ketamine exert their anesthesia effects through antagonizing NMDA receptor. But it is reported that ketamine-induced psychopathology results from glutamate increase in anterior cingulate cortex (7). The neuronal hyperactivity in the cingulate cortex and the thalamic nuclei was believed to relate to ketamine-induced psychotomimetic effects, which was proved by high expression of c-Fos in these areas (8, 9). The increase glutamate was thought to inhibit GABAergic [ability to produce gamma-aminobutyric acid (GABA)] neurons through activation of interneurons. The drug which can activate GABA receptors can be used to reduce esketamine-induced psychoactive side effects. Therefore, esketamine or ketamine was usually used with midazolam, propofol, and thiopental to decrease psychoactive effects (7). Midazolam is widely used in the clinical practice such as acute epileptic seizures and electrical defibrillation (10, 11). Esketamine and midazolam (Esk+Mida) can be used together to achieve an anesthetic effect and decrease psychotomimetic adverse effects (12, 13). However, it was not known whether midazolam could efficiently attenuate esketamine-induced overactive behaviors and neuronal hyperactivities. The purpose of this study was to determine whether midazolam could inhibit the central nervous excitation of esketamine through behaviors observation and detection of c-Fos expression in mice brain. The optimal dose of midazolam was also investigated when it was administrated with esketamine. We hypothesized that midazolam could efficiently prevent esketamine-induced psychotomimetic effects when using together.

## Materials and Methods

### Ethics Statement

This experiment was approved by the ethics committee of the Zhengzhou Central Hospital. All the processes were strictly adhered to the Chinese Animal Welfare Act.

### Animals

The male Kunming mice weighing around 25 g were purchased from the Animal Center of the Medical College of Zhengzhou University. Every 2–3 mice were allocated to a cage, where the ambient temperature was 22 ± 2°C, humidity was maintained at about 40 ± 5%, and sufficient food and water were ensured with wood bedding in accordance with the Chinese guidelines. Before the experiment, each mouse was acclimated to the testing room for 24 h. The mice were randomly divided into the different groups. Adequate oxygen supply was ensured during medication treatment.

### Drugs

The doses of esketamine (Hengrui Medicine, Lianyungang, China) were 50 mg/kg (14), and the administration dose of midazolam were 40 mg/kg, 80 mg/kg, 120 mg/kg (Hengrui Pharmaceutical, Jiangsu, China). According to the calculated dose in advance, the drugs were diluted to different concentration gradients with normal saline to ensure injection of the same volume (1 ml), and the saline group was injected with the same volume of 1 ml normal saline. The intraperitoneal injection was performed by inserting the needle to its full length at the lower right quadrant to head with 30–40 angles to horizon. Aspiration without blood during injection should be confirmed to avoid intravascular administration.

### Experimental Design

The experimental mice were randomly divided into the following groups in which the treatment details were descripted in Table 1.

**Table 1:** The details of the designed groups in this study.

**Groups**	**Sample size**	**Treatment (intraperitoneal injection)**
Saline	*n =* 9^#^	Saline
Esk	*n =* 9^#^	Esketamine 50 mg/kg
Mida40	*n =* 8^&^	Midazolam 40 mg/kg
Mida80	*n =* 7^@^	Midazolam 80 mg/kg
Mida120	*n =* 6^!^	Midazolam 120 mg/kg
Esk+Mida40	*n =* 6	Esketamine 50 mg/kg+Midazolam 40 mg/kg
Esk+Mida80	*n =* 8	Esketamine 50 mg/kg+Midazolam 80 mg/kg
Esk+Mida120	*n =* 8	Esketamine 50 mg/kg+Midazolam 120 mg/kg

*The recovery time varied extremely among the groups. Esk+Mida group extended the recovery time when compared with Esk or the corresponding Mida groups. To make the expression of c-Fos in brain comparable, the brains from each group should be retrieved at the same time after the drug administration*.

*^#^3 mice from both Saline and Esk group were matched to the mice from each Esk+Mida group to retrieve brain at the same time after full recovery to detect c-Fos expression*.

*^&^The mice from Mida40 group were matched to Esk+Mida40 group to retrieve brain at the same time after full recovery*.

*^@^The mice from Mida80 were matched to Esk+Mida80 groups to retrieve brain at the same time after recovery*.

*^!^The mice from Mida120 were matched to Esk+Mida120 groups to retrieve brain at the same time after recovery*.

### Experimental Procedure

During the experiment, each mouse was placed separately in a cage and given 100% pure oxygen to observe and record any changes in behaviors before and after the administration of the drug. The behaviors registered were agitation, sedation, pedal withdrawal reaction (PWR), and righting reaction (RR). Agitation was characteristic by involuntary myoclonic twitching, running aimlessly, stiffness, loss of coordination and balance in walking, and inability to keep normal posture. Sedation was characterized by a stage of immobilization, sleep-like posture, but still responsive to stimulation. PWR was recorded when mice attempt to withdraw the limb to toe pinch (14). The pinch was performed by a small tweezers without teeth and should not cause any injuries in the toes. Loss of PWR was determined by the disappearance of reactions to all the three consecutive pinches. The mice regained PWR when they could respond to toe pinch for all the three times. The mouse was physically rolled into lateral recumbency by hand to evaluate RR (15, 16). The mouse with normal RR would be returned to ventral recumbent position within 10 s. Loss of RR was recorded when the mouse was unable to right their posture for all the three times. The mice regained RR when they were able to right their posture for all the three times. Anesthesia state was defined as the duration of loss of PWR and loss of RR. These behaviors were continuously monitored for up to 4 h.

Action time was defined as the time from the injection drug to emergence of any signs of ataxia, agitation, or sedation (14, 17). Duration of agitation before the sedation was regarded as the cumulative time from the beginning of the abnormal behaviors to the start of sedation (14). Duration of sedation was defined as the cumulative time of sedation. Duration of loss of PWR was defined as the time from the first time that three consecutive effective stimuli disappeared to the time of the response to all the three stimulus recovered. Duration of loss of RR was defined as the cumulative time of loss of RR. Recovery time was defined as the time from the action starts to the animal shows normal behaviors including regaining all of RR, PWR, and coordination together. Duration of agitation during the recovery was regarded as the cumulative time of agitation from the end of sedation to the time of recovery.

After the full recovery from anesthesia, the mice were euthanized by decapitation under anesthetized by chloral hydrate. The mice brains were retrieved for western blot and immunohistochemistry (IHC).

### Immunohistochemistry

Mice brains were put into 4% paraformaldehyde for at least 24 h at 4°C. The brains were embedded in paraffin after dehydration and clearance. The paraffin-embedded tissue blocks were cut into sections at 4 μm thickness on a microtome. Sections on glass slides were deparaffinized and rehydrated before immunohistochemistry. Sections were boiled in citrate buffer (pH 6.0) for 20 min for antigen retrieval. The endogenous peroxidase was blocked by incubating in 3% H_2_O_2_ solution for 10 min at room temperature. The primary antibody c-Fos (Bioss, China) was incubated with the sections over night at 4°C. After washing, the biotinylated secondary antibody and streptavidin-HRP were sequentially incubated with the sections. After washing, the sections were stained using DAB substrate solution (Zsbio, China). The sections were counterstained by hematoxylin. Then, the sections were dehydrated and cleared before mounting coverslips. Finally, sections were visualized under a microscope at × 400 or × 100 (Olympus, Japan).

### Western Blot

The retrieved mouse brains were stored at −80°C and the proteins were extracted using the RIPA buffer (Epizyme, China). The concentration of the extracted protein was determined using the BCA protein assay kit (Epizyme, China). The same amounts of protein were run on 10% sodium dodecyl sulfate polyacrylamide gel electrophoresis (SDS-PAGE) and transferred onto polyvinylidene fluoride (PVDF) membranes. The membranes were incubated in 5% non-fat milk for 1 h at room temperature. The membranes and the primary antibodies c-Fos (Bioss, China) were incubated together and overnight at 4°C, and then incubated with an HRR-conjugated secondary antibody (Abbkine, China). The GADPH protein (Boster, China) serves as the internal reference protein. The proteins expression was analyzed using an ESL scanner with Omni-ECL Enhanced Pico Light Chemiluminescence kit (Epizyme Biotech, China), and signal intensities of the protein expression were quantified using Chemidoc EQ system (BioRad, USA).

### Statistical Analysis

The sample size was calculated according to duration of agitation before the sedation which was attained from our previous paper (18). The sample size was set to at least 6 per group using formula sample size = 2 SD^∧^2 (Z^∧^(α/2) + Z^∧^β)^∧^2/d^∧^2 (19). Some groups had more than 6 mice because c-Fos expression in brain was detected to match each group. Behavior data presented in this study are described as median with 25–75 percentiles. Median analysis was performed using the Kruskal-Wallis rank sum test followed by the Conover's *post-hoc* test. The *P*-value is adjusted according to the family-wide error rate (FWER) procedure of holm, and then by the false discovery rate (FDR) procedure of Benjamini-Hochberg. Western blot results were presented as mean ± SD. ANOVA was used to analyze the c-Fos expression differences. *P* < 0.05 was considered as significant difference.

## Results

### General Behaviors After the Intraperitoneal Administration of Esketamine and Midazolam

After injection of esketamine, the mice gradually appeared ataxia including loss of coordination in walking, imbalance, inability to keep posture. Subsequently, the mice displayed agitation, some of them catalepsy, which demonstrated immobilization associated with intensive muscle tone all over body, like arched-back rigidity. After excitation and catalepsy, the mice returned to ataxia again and regained coordination and balance gradually.

### Action Time

**Figure 1:**
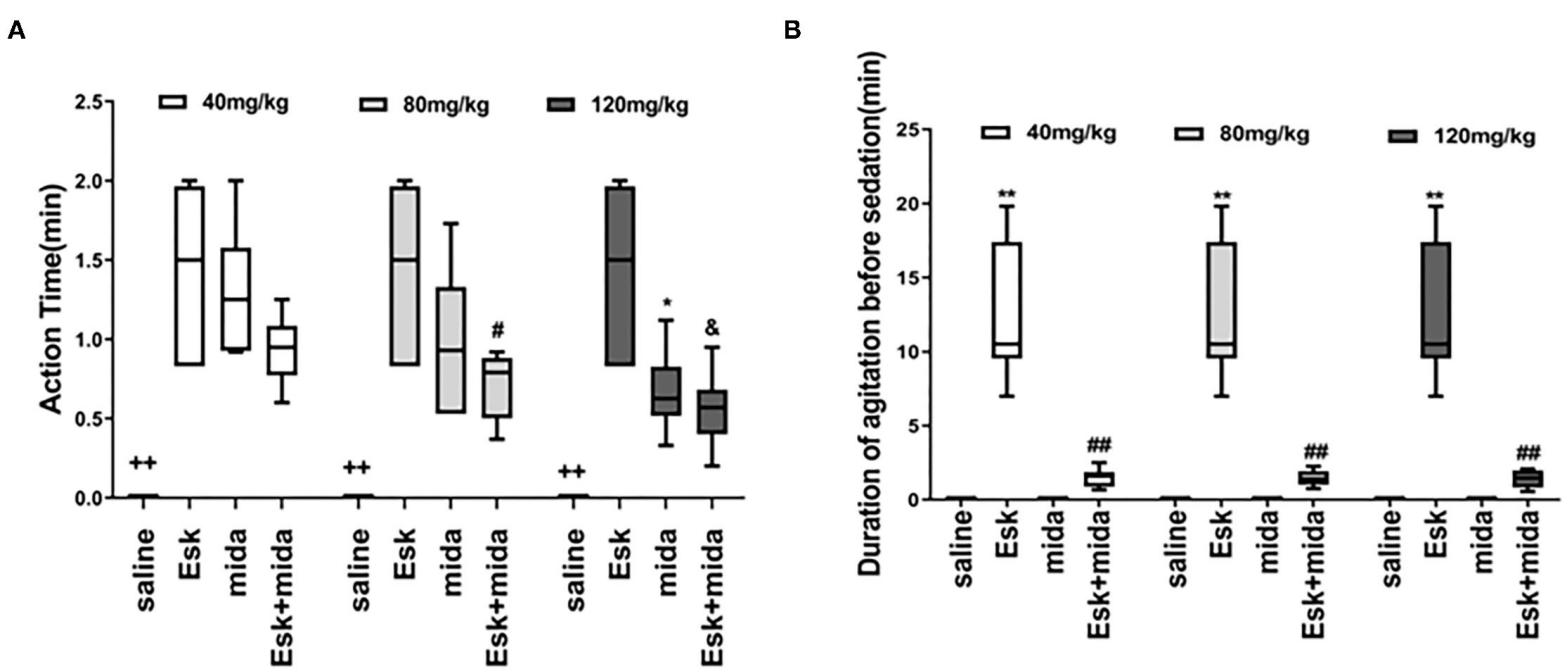
Action time and recovery time. **(A)** Action time ^++^*P* < 0.01, compared to Esk, Mida, and Esk+Mida groups. ^#^*P* < 0.05, compared to Esk group. ^*^P < 0.05, compared to Esk group. ^&^*P* < 0.05, compared to Esk group. **(B)** Duration of agitation before the sedation. ^**^P < 0.01, compared to Saline, Mida, and Mida + Esk groups. ^*##*^*P* < 0.01, compared to Saline and Mida groups.

The median of action time was about 1.5 min for esketamine. There is a significant difference between Mida120 and Esk groups. Esk+Mida80 and Esk+Mida120 groups had a shorter action time than Esk groups. However, these differences were not clinically significant because all action times were within 2 min (Figure 1A, Table 2).

**Table 2:** Results of behaviors observation [median (25percentiles, 75percentiles)].

**Measures**	**Saline**	**Esk**	**Mida40**	**Mida40+Esk**	**Mida80**	**Mida80+Esk**	**Mida120**	**Mida120+Esk**
	**(*n* = 9)**	**(*n* = 9)**	**(*n* = 8)**	**(*n =* 6)**	**(*n* = 7)**	**(*n* = 8)**	**(*n* = 6)**	**(*n* = 8)**
Action time (min)	0	1.50 (0.83, 1.965)^++^	1.25 (0.928, 1.578)^++^	0.95 (0.773, 1.085)^++^	0.93 (0.53, 1.33) ^++^	0.79 (0.503, 0.883)^++#^	0.625 (0.518, 0.828) ^++*^	0.57 (0.403, 0.683)^++&^
Duration of agitation before sedation (min)	0	10.51 (9.56,17.39)**	0	1.60 (0.895, 1.878)^*##*^	0	1.405 (1.02, 1.933)^*##*^	0	1.475 (0.835, 1.978)^*##*^
Duration of sedation (min)	0^++^	0**	53.87 (33.25, 56.56)	38.42 (26.11, 52.85)	58.33 (53, 77.67)^&&^	105.5 (85.46, 122.8)^*##*^	126.3 (91.96, 148.8)^@@^	145.2 (120.6, 156.9)^∧∧^
Duration of Loss of RR (min)	0^++^	5.2 (0.49, 6.94)**	0^∧∧^	50.71 (39.69, 84.77)	0^∧∧^	114.4 (91.99, 122.0)^&&^	9.3 (0, 32.50)^∧∧*##*^	148.5 (130.0, 167.0)^&&^
Duration of Loss of PWR (min)	0	5.200 (0, 5.69)	0	44.46 (32.44, 60.32)^*##*^	0	106.6 (91.39, 118.6)^##^	7.99 (0, 20.55)	154.2 (137.3, 167.0) ^*##*^
Duration of agitation in recovery (min)	0	0	0	38.85 (24.00, 49.00)^*##*^	0	36.00 (26.25, 44.50)^##^	0	46.00 (27.00, 59.25) ^*##*^
Recovery time (min)	0^++^	28.07 (22.40,30.50)^∧∧^	56.65 (34.06,59.25)^##^	76.51 (60.48,90.09)	59.07 (54.00,77.83)^*##*^	153.0 (89.49,174.6)	128.5 (93.10,150.5)^*##@@*^	192.6 (179.9,210.7)**

Action Time: ^++^P < 0.01, compared to saline groups. ^#^P < 0.05, compared to Esk group. ^*^P < 0.05, compared to Esk group. ^&^P < 0.05, compared to Esk group.

Duration of agitation before the sedation: ^**^P < 0.01, compared to Saline, Mida, and Mida+ Esk groups. ^##^P < 0.01, compared to Saline and Mida groups..

Duration of sedation: ^++^P < 0.001, compared to Mida and Esk+Mida groups. ^**^P < 0.001, compared to Mida and Esk+Mida groups. ^##^P < 0.01, compared to Esk+Mida40. ^∧∧^P < 0.01, compared to Esk+Mida40 group. ^&&^P < 0.01, compared to Mida40 group. ^@@^P < 0.01, compared to Mida80 and Mida40 group..

Duration of RR: ^++^P < 0.01, compared to Esk+Mida groups. ^**^P < 0.01, compared to Esk+Mida groups. ^∧∧^P < 0.01, compared to Esk+Mida40, Esk+Mida80 and Esk+Mida120 group. ^&&^P < 0.01, compared to Esk+Mida40 group. ^##^P < 0.01, compared to Mida40 and Mida80 groups..

Duration loss of PWR: ^##^P < 0.01, compared to Esk, Mida, and Esk+Mida groups. ^**^P < 0.01, compared to Esk groups..

*Duration of agitation during the recovery: ^##^P < 0.01, compared to Saline, Mida, and Esk groups.*.

Recovery Time: ^++^P < 0.01, compared to Esk, Mida, and Mida+Esk groups. ^∧∧^P < 0.01, compared to Mida, and Esk+Mida groups. ^##^P < 0.01, compared to Esk+Mida group. ^@@^P < 0.01, compared to Mida40 and Mida80. ^**^P < 0.01, compared to Esk+Mida40.

### Duration of Agitation Before the Sedation

The agitation before sedation was found in esketamine and Esk+Mida groups. The durations of agitation before sedation in all Esk+Mida groups were shorter than that of Esk group. There were no differences in the duration of agitation before the sedation among Esk+Mida groups. The duration of agitation before the sedation was transient and lasted <3 min in all Esk+Mida groups (Figure 1B, Table 2).

### Duration of Sedation

**Figure 2:**
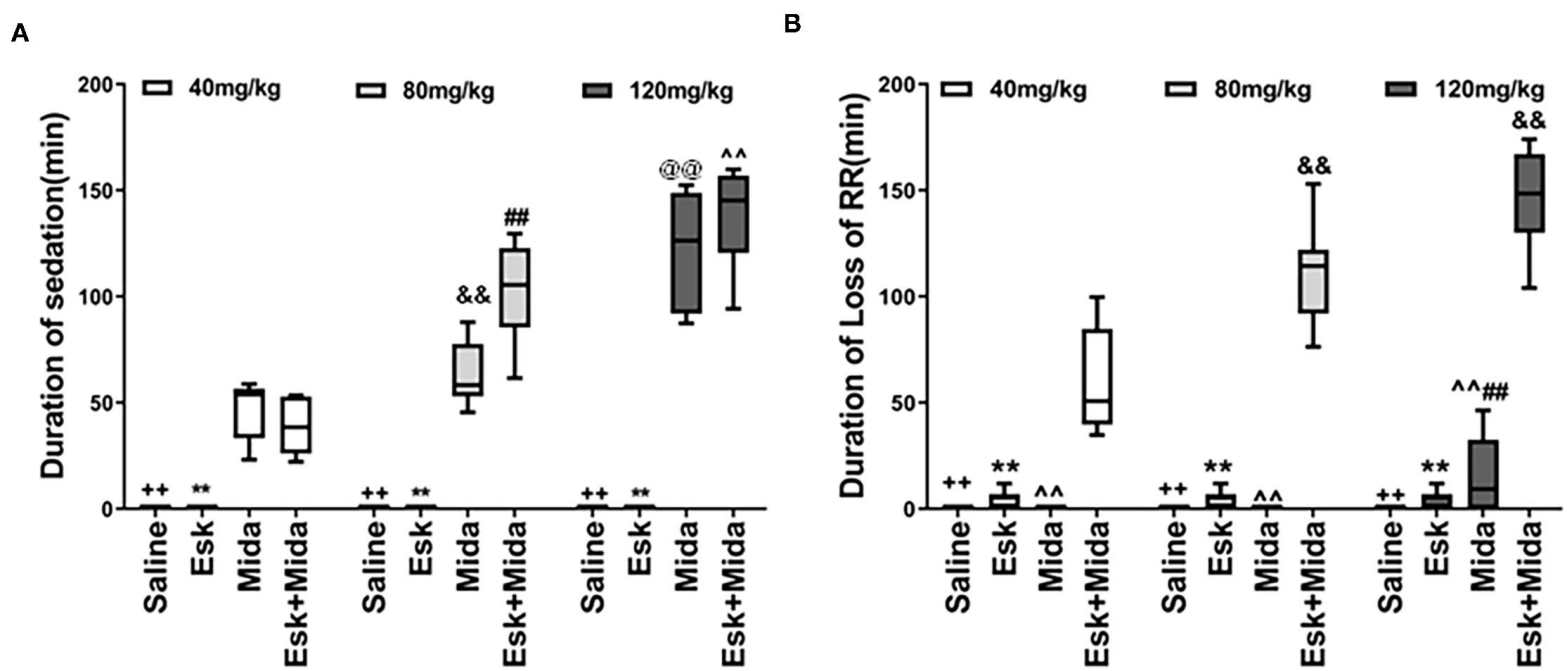
Duration of sedation and loss of RR. **(A)** Duration of sedation. ^++^*P* < 0.001, compared to Mida and Esk+Mida groups. **P < 0.001, compared to Mida and Esk+Mida groups. ^##^*P* < 0.01, compared to Esk+Mida40. ^∧∧^*P* < 0.01, compared to Esk+Mida40 group. ^&&^*P* < 0.01, compared to Mida40 group. ^@@^*P* < 0.01, compared to Mida80 and Mida40 groups. **(B)** Duration of RR. ^++^*P* < 0.01, compared to Esk+Mida groups. **P < 0.01, compared to Esk+Mida groups. ^∧∧^*P* < 0.01, compared to Esk+Mida40, Esk+Mida80 and Esk+Mida120 groups. ^&&^*P* < 0.01, compared to Esk+Mida40 groups. ^##^*P* < 0.01, compared to Mida40 and Mida80 groups.

There was almost no sedation in esketamine group. Midazolam dose-dependently increased duration of sedation when used alone or with esketamine (Figure 2A, Table 2).

### Duration of Loss of RR

There were no differences in duration of loss of RR between esketamine and midazolam groups. However, the durations of loss of RR were extended significantly when combination of esketamine and midazolam were administrated. Esk+Mida40 group showed a shorter duration of loss of RR than Esk+Mida80 or Esk+Mida120 groups. There were no differences in duration of loss of RR between Esk+Mida80 and Esk+Mida120 groups (Figure 2B, Table 2).

### Duration of Loss of PWR

**Figure 3:**
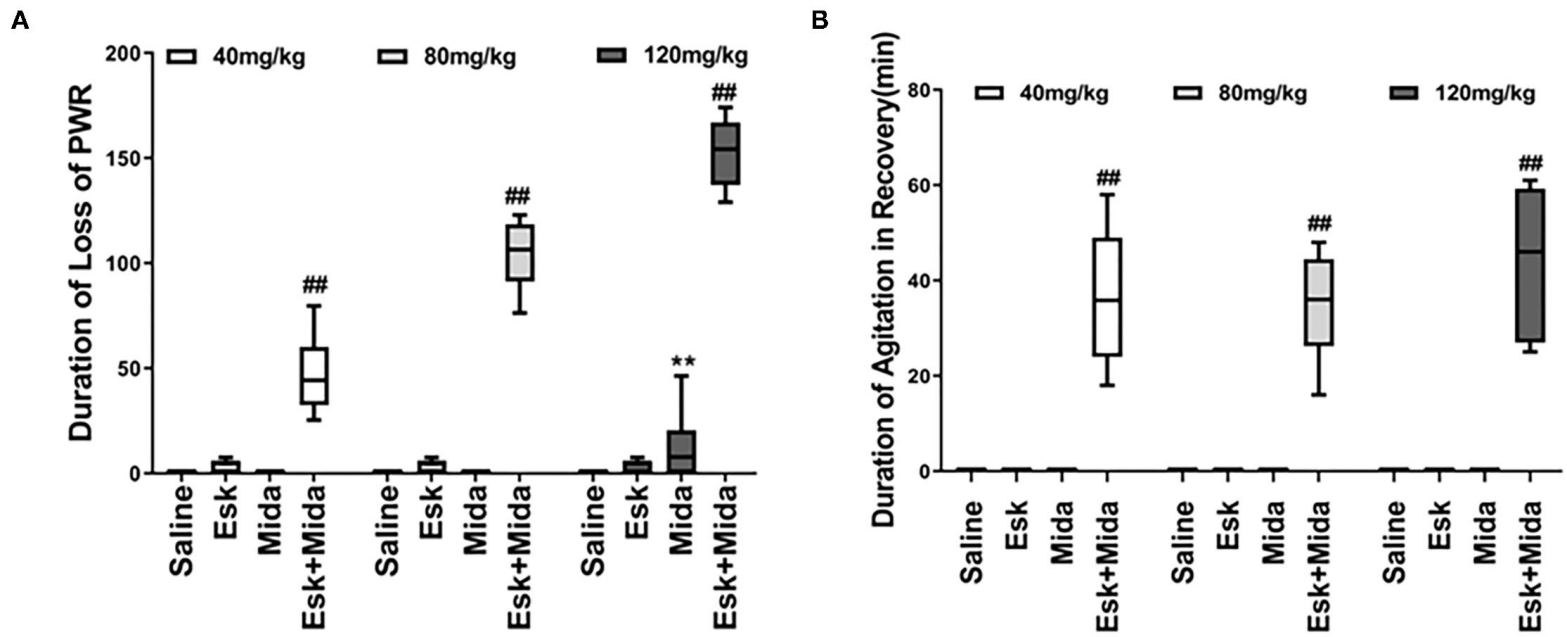
Duration of loss of PWR and duration of agitation during the recovery after injection. **(A)** Duration loss of paw withdraw reaction. ^##^*P* < 0.01, compared to Esk, Mida, and Esk+Mida groups. **P < 0.01, compared to Esk groups. **(B)** Duration of agitation during the recovery. ^##^*P* < 0.01, compared to Saline, Mida, and Esk groups.

Esk, Mida40, and Mida80 did not have significant effects on PWR. Only Mida120 could extend PWR a little longer than Mida40 or Mida80 groups. However, the durations of loss of PWR were extended significantly when combination of esketamine and midazolam were administrated. Esk+Mida40 group showed a shorter duration of loss of PWR than Esk+Mida80 or Esk+Mida120 groups. Esk+Mida80 group had a shorter duration of loss of RR than Esk+Mida120 group (Figure 3A, Table 2).

### Duration of Agitation During the Recovery

The agitation during the recovery was found only in combination of esketamine and midazolam. There is no agitation during recovery in Esk group because esketamine did not induce any sedation (Figure 3B, Table 2). Duration of agitation during the recovery ranged from 20 to 75 min in all the three Esk+Mida groups. There were no differences in the duration of agitation during the recovery among Esk+Mida40, Esk+Mida80, and Esk+Mida120. The start times of the recovery agitations of Esk+Mida40, Esk+Mida80, and Esk+Mida120 groups were about 60, 100, and 150 min, respectively, after administration of the mixed fluid.

### Recovery Time

**Figure 4:**
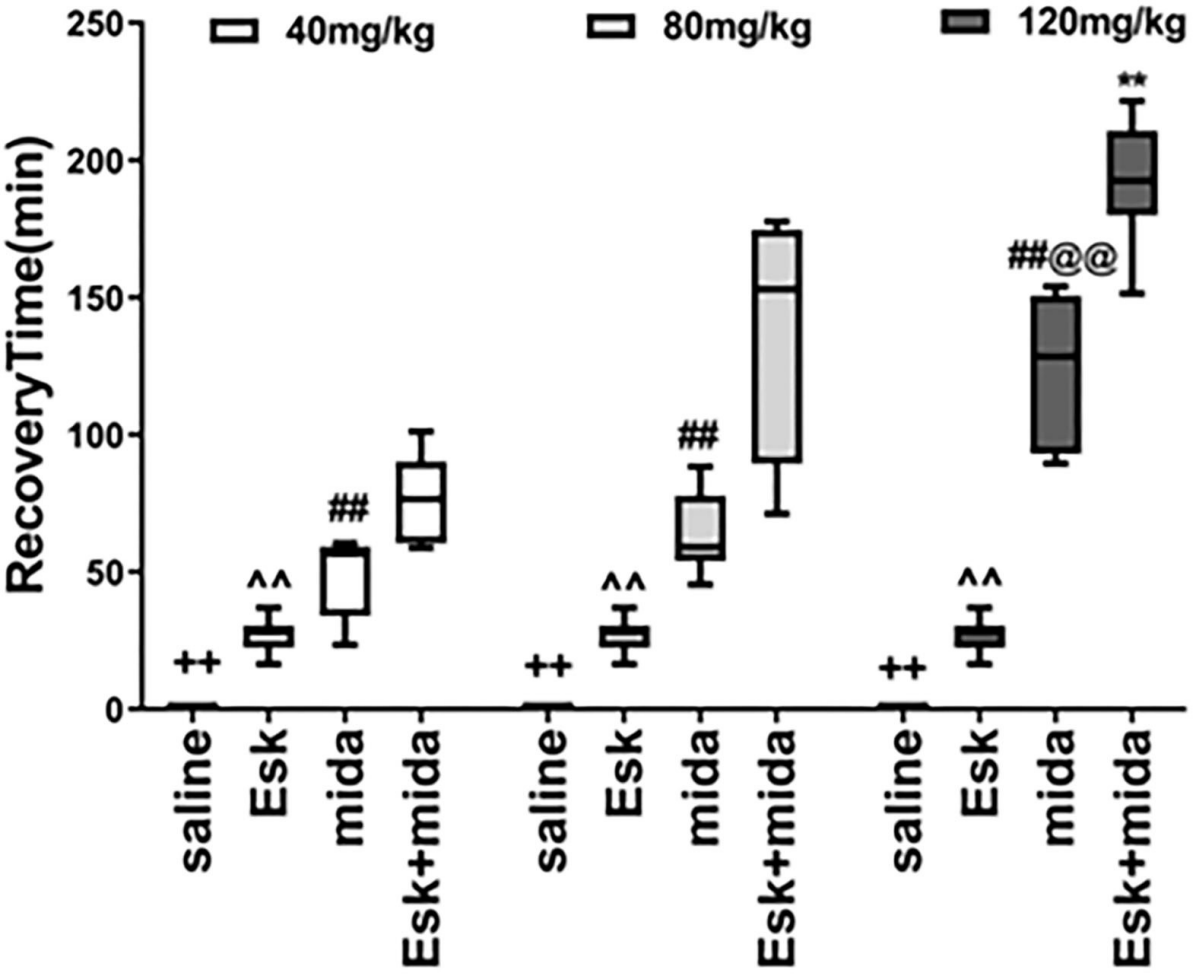
Recovery Time. ^++^*P* < 0.01, compared to Esk, Mida, and Mida+Esk groups. ^∧∧^*P* < 0.01, compared to Mida, and Esk+Mida groups. ^##^*P* < 0.01, compared to Esk+Mida group. ^@@^*P* < 0.01, compared to Mida40 and Mida80 groups. **P < 0.01, compared to Esk+Mida40 groups.

The median of recovery time of esketamine was 30 min which was less than all midazolam and Esk+Mida groups. The recovery time of Mida120 group was longer than that of Mida40 and Mida80 groups. There were no differences in recovery time between Mida40 and Mida80 groups. The Esk+Mida groups had a longer recovery time than the corresponding Mida groups. Midazolam dose-dependently increased recovery times in Esk+Mida groups (Figure 4, Table 2).

### Anesthesia Stage

Because anesthesia stage was defined as the duration of loss of both PWR and RR in this study, anesthesia stage could be identified as the lower duration from the duration of loss of PWR and RR. Therefore, the results regarding anesthesia stage are available in duration of loss PWR and RR.

### C-Fos Expression in Mice Brain

**Figure 5:**
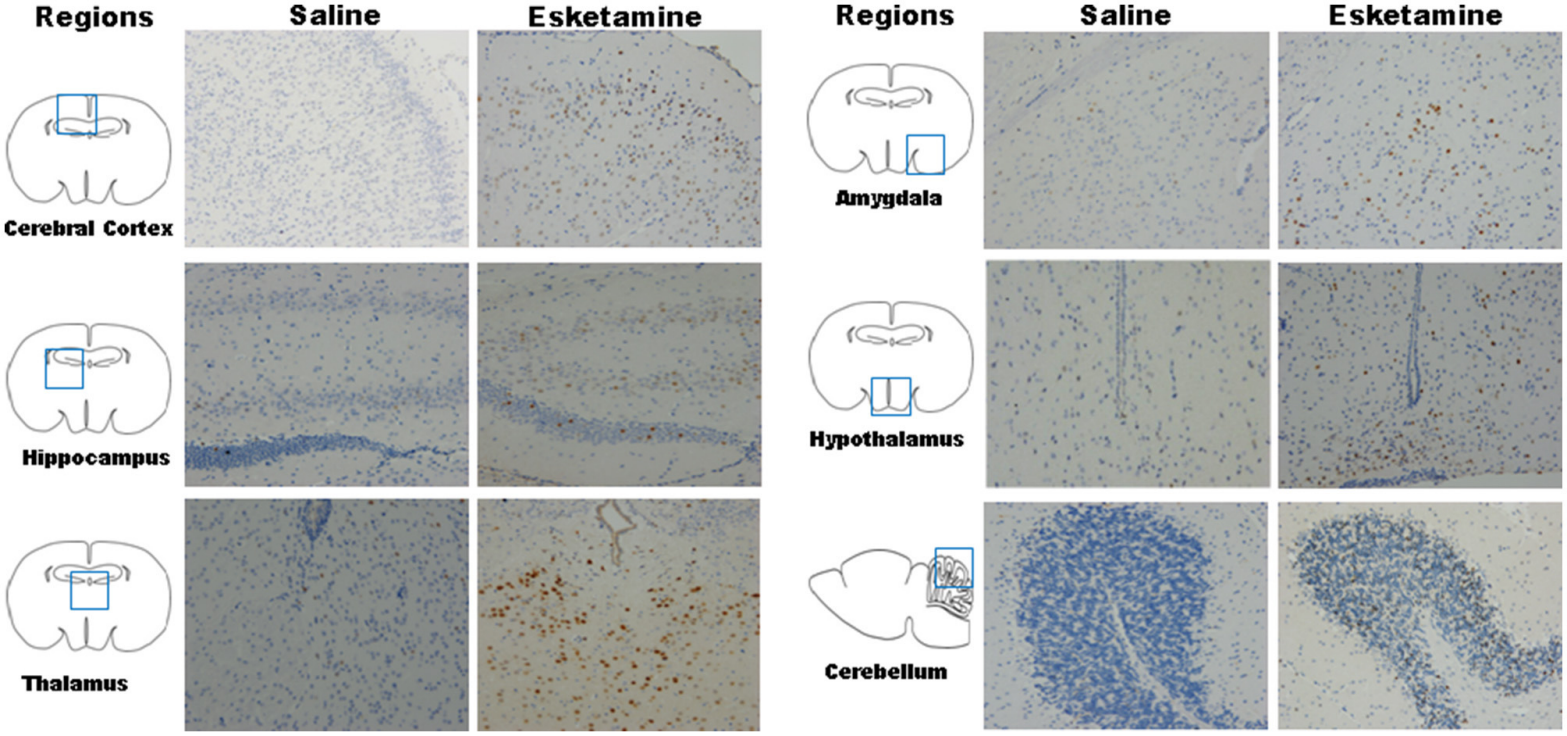
c-Fos expression in mice brain regions including cerebral cortex, hippocampus, thalamus, amygdala, hypothalamus, and cerebellum after recovery (× 200).

Esketamine group had more c-Fos-positive neurons in a high-power field under microscope than saline or midazolam groups in the mice brain, including cerebral cortex, hippocampus, thalamus, amygdala, and hypothalamus. However, All Mida +Esk groups demonstrated the similar numbers of c-Fos-positive neurons as the esketamine group (Figure 5).

### Western Blot Result

**Figure 6:**
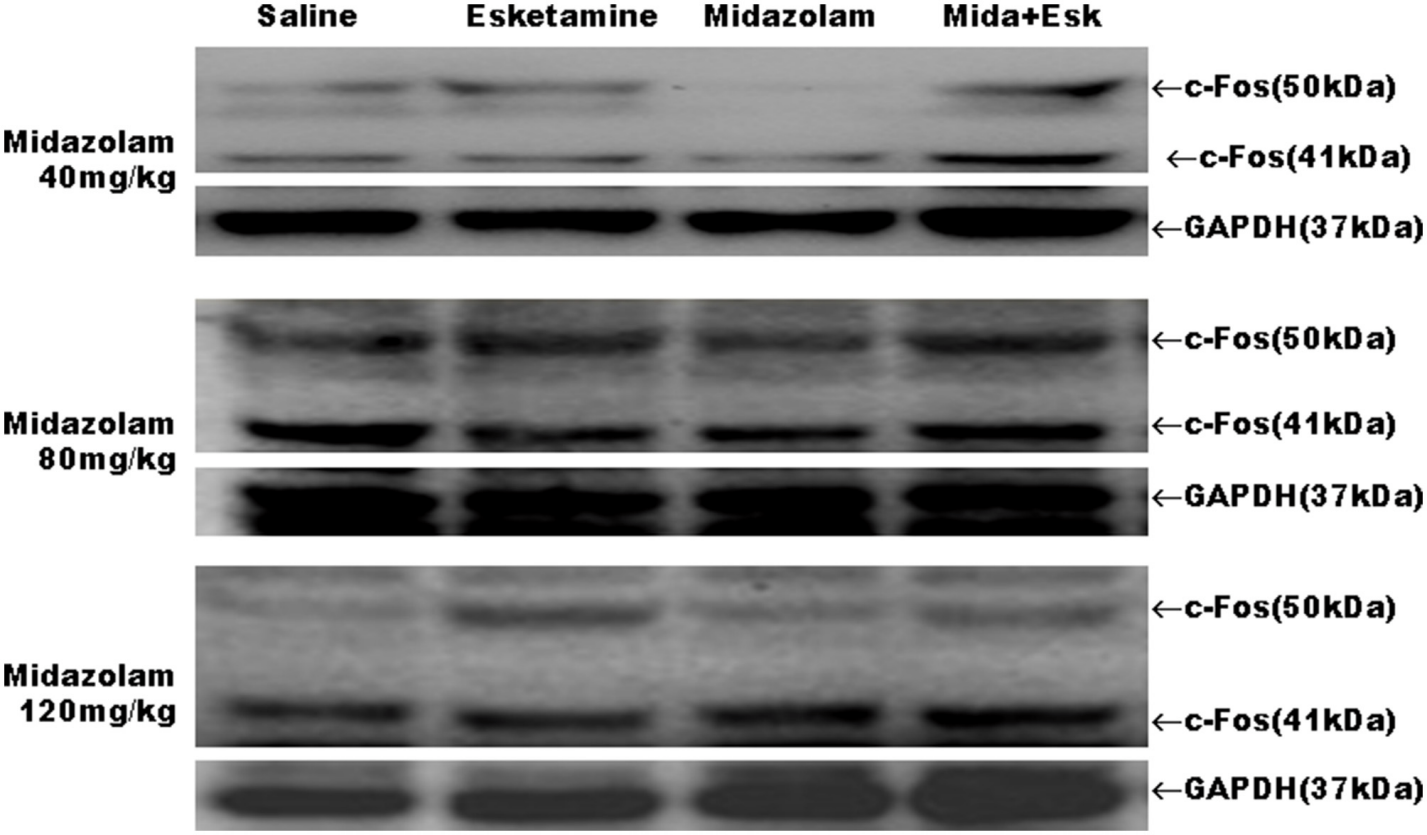
Representative western blot results for c-Fos expression in mice brain.

**Figure 7:**
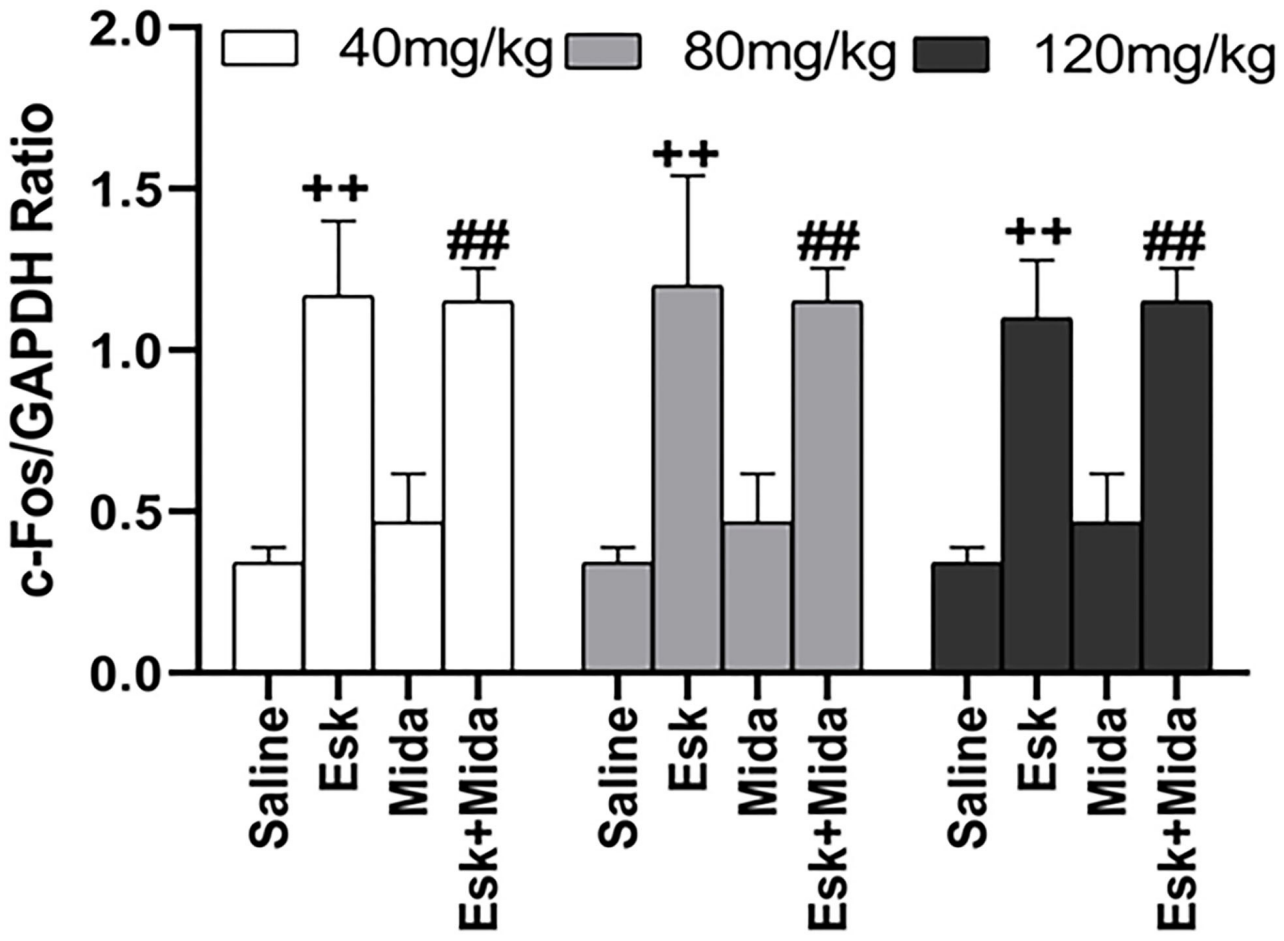
Groups analysis of c-Fos expression over GAPDH ratio in mice brain after recovery from combination of esketamine and midazolam. ^++^*P* < 0.01, compared to saline group. ^##^*P* < 0.01, compared to Mida group.

Midazolam at 40, 80, and 120 mg/kg had no effects on esketamine-induced c-Fos expression (Figures 6, 7).

## Discussion

In this study, we found that midazolam decreased esketamine-induced agitation before the sedation. However, midazolam had no effects on the agitation during the recovery. Midazolam dose-dependently extended sedation and recovery time not only alone but also combined with esketamine. Combination of esketamine and midazolam synergically enhanced each other by increasing duration of loss of RR and PWR to get an anesthesia state which were not reached by either esketamine or midazolam alone.

Psychoactive effects were the main concern for esketamine or ketamine in anesthesia (20). Because ketamine and midazolam had similar half-life time for pharmacokinetics (21, 22), they are usually combined in animal and human anesthesia to decrease psychoactive effects and improve the quality of anesthesia (12, 13, 23). Midazolam exerts its function by stimulation of the γ-aminobutyric acid (GABA) receptors in the central nervous system, which leads to a postsynaptic increase in chloride ions, thereby reducing neuronal excitability (24). Therefore, it is an advantage for esketamine or ketamine to use with the GABA receptor agonist to decrease the psychoactive side effects and to sustain the respiratory function during anesthesia (25). Our study demonstrated that combination of midazolam and esketamine avoid agitation before sedation and an anesthesia state can be achieved by the synergism.

The aim of this study was to determine if midazolam could inhibit the central nervous excitation of esketamine through behaviors observation. One of these behaviors was agitation, characterized by ataxia, aimless running, myoclonic twitching, catalepsy, and overactive behaviors. Because these behaviors changed too fast to record, agitation was used to include all these overactive behaviors. We found that the agitations before sedation were attenuated efficiently by midazolam in mice. It is consistent with our hypothesis that midazolam can suppress esketamine-induced overactive behaviors as dexmedetomidine (18).

To our surprise, midazolam could not decrease the agitation during the recovery. At first, we expected that the agitation can disappear when the dose of midazolam was increased. However, high doses of midazolam still had no effects on the duration of agitation during the recovery. There were no significant differences in the duration of recovery agitation among 40, 8, and 120 mg/kg midazolam with esketamine. The duration of recovery agitation among these groups ranged from 20 to 75 min. Comparing to the esketamine-induced agitation in Esk group, the duration of the recovery agitation was longer (10–20 vs. 20–75 min) in Esk+Mida groups, but the intensity of agitation was milder according to the subjective observation. It was reported that mice would experience ataxia during the recovery from anesthesia of s-ketamine and medetomidine (10). The mice presented ataxia up to 2 h after medetomidine was antagonized by atipamezole. This symptom was consistent with our findings in this study.

Because the agitation before sedation is different from the agitation during recovery in duration and intensity, c-Fos expression was measured using western blot and IHC to confirm whether both of them come from neuronal hyperactivities (26). Our results indicated that there were no differences in c-Fos expression between Esk and Esk+Mida groups. It suggested that both the agitation from esketamin alone and the agitation during the recovery in Esk+Mida come from neuronal hyperactivities.

Why did midazolam have no effects on the recovery agitation in combination with esketamine? The obvious reason may be that midazolam has a shorter effect than esketamine. However, midazolam 120 mg/kg extended the duration of sedation by 3 times that of 40 mg/kg midazolam, the recovery agitation still started after 2–3 h delay and lasted for the similar range of duration. The possible reason may be that the recovery agitations come from the metabolite of esketamine (27–29). Ketamine can be converted to an active metabolite, norketamine, by CYP3A4 (27). Norketamine, included Nor-S-ketamine and Nor-R-ketamine can retain anesthetic effects, which can explain the maintenance of the anesthesia at lower blood ketamine concentrations (30, 31). Norketamine can also cross the blood–brain barrier and induces psychoactive effects (23, 32). This norketamine-induced psychoactive effects show mild when compared to esketamine or ketamine-induced agitation because the efficacy of norketamine is less powerful than that of ketamine. The elimination of norketamine need a longer time than ketamine (t_1/2_ 1.5 h vs. 30 min). This longer elimination time may account for the recovery agitation after a long sedation of high dose of midazolam in this study (6, 23, 33).

There are several limitations in this study including small sample size, lots of subjective data, and non-specific indicator for neuronal activity. First, the sample size was just set to detect the difference in duration of agitation before the sedation. So, this study may have no enough power to find any other difference. Second, all the behaviors data were attained through observation, which is easy to be affected by observer's subjectivity. Third, c-Fos was used to reflect neuronal activity in this study. However, c-Fos expression is very sensitive to any stimulation and is not specific to reflect neuronal activity induced by esketamine. Last, only male mice were chosen in this study. The findings will not be generalized to both male and female when combination of midazolam and esketamine was applied in anesthesia.

## Conclusion

Midazolam 40, 80, and 120 mg/kg efficiently decreased the esketamine-induced overactive behaviors including ataxia, excitation, and catalepsy before sedation. Midazolam and esketamine synergically improved the anesthesia quality assessed by PWR and RR. However, even high doses of midazolam were not able to suppress the excitatory effects of esketamine during the recovery. It suggested that it is not an ideal strategy to administrate one dose of midazolam to minimize esketamine-induced psychoactive effect in anesthesia. A further study was warranted to set up a continuous or multiple administration method to combine midazolam with esketamine in animal anesthesia.

## Data Availability Statement

The raw data supporting the conclusions of this article will be made available by the authors, without undue reservation.

## Ethics Statement

The animal study was reviewed and approved by the Ethics Committee of Zhengzhou Central Hospital. All processes were strictly adhered to the Chinese Animal Welfare Act.

## Author Contributions

QC prepared and wrote the manuscript, performed data analysis, and contributed to manuscript review and preparation. MM collected data, prepared tables and figures, and contributed to manuscript review and preparation. YB aided in the study design, collected and analyzed data, and contributed to manuscript writing and review. LS prepared and performed IHC experiment. DZ contributed to the experiment of western blot. PZ contributed to article writing and data analysis. XJ designed the study, performed data analysis, contributed to manuscript writing and review, and supervised all aspects of the study. All authors read and approved the final manuscript.

## Conflict of Interest

The authors declare that the research was conducted in the absence of any commercial or financial relationships that could be construed as a potential conflict of interest.

## Publisher's Note

All claims expressed in this article are solely those of the authors and do not necessarily represent those of their affiliated organizations, or those of the publisher, the editors and the reviewers. Any product that may be evaluated in this article, or claim that may be made by its manufacturer, is not guaranteed or endorsed by the publisher.

## Abbreviations

Mida40: Midazolam 40 mg/kg
Mida80: Midazolam 80 mg/kg
Mida120: Midazolam 120 mg/kg
Esk+Mida40: Esketamine 50 mg/kg + Midazolam 40 mg/kg
Esk+Mida80: Esketamine 50 mg/kg + Midazolam 80 mg/kg
Esk+Mida120: Esketamine 50 mg/kg + Midazolam 120 mg/kg
Esk+Mida, including Esketamine 50 mg/kg + Midazolam 40 mg/kg, Esketamine 50 mg/kg + Midazolam 80 mg/kg: and Esketamine 50 mg/kg + Midazolam 120 mg/kg
PWR: Pedal Withdrawal Reaction
RR: Righting Reflex.
